# Supplementary material for: The asynchronous establishment of chromatin 3D architecture between in vitro fertilized and uniparental preimplantation pig embryos
Source: Genome Biol. 2020 Aug 10;21:203. doi: 10.1186/s13059-020-02095-z (PMC7418210; doi:10.1186/s13059-020-02095-z)
Supplement: Supplementary file 1 — Additional file 1. Supplementary Text. [file 13059_2020_2095_MOESM1_ESM.docx]

**Supplementary Text**

**CTCF chromatin loops are gradually established in pig preimplantation embryos but not in mouse.**

At chromatin loop level, we found that a small fraction of CTCF chromatin loops in PEF may be established in pig preimplantation embryos, while little such loops in MEF has been established before ICM stage in mouse. In mammals, about ~39% of the TAD boundaries carry strong chromatin loops, which are stable for binding of structural proteins like CTCF [[9](#_ENREF_9)]. As TADs are believed to be conserved between cell types [[9](#_ENREF_9)], these strong and stable CTCF-anchored chromatin loops should also be conserved. Thus, we set out to determine how those loops are established in pig preimplantation embryos. We used the CTCF loops in PEFs as a reference and compared the aggregate peak analysis (APA) scores between zygote, 4-cell and morula stages (Figure S5A). The APA scores are all gradually increased in the three lineages with IVF be the fastest (Figure S5A and B). At the zygote stage, the z-scores are negative, indicating a depletion of CTCF-anchored chromatin loops. The loops seem to be established at the 4-cell stage as indicated by positive z-scores, and the loop strength continues to grow at the morula stage. However, none of the z-scores in the morula stage is comparable to that in PEF stage (Figure S5B). Even for IVF, the z-score is 4.884 at the morula stage, while the score is 36.253 in PEFs. On the other hand, remarkably different patterns are seen in mouse preimplantation embryos, compared with pig. Using the CTCF-anchored chromatin loops in MEFs as a reference, the z-scores remain negative even at the ICM stage with z-scores equal to -0.405, 0.094 and -0.935 for total, maternal and paternal chromosomes, respectively (Figure S5C). These results indicate that initiation of CTCF-anchored chromatin loops may not even started as late as the mouse ICM stage. The early initiation of chromatin loop establishment during ZGA in pig may not necessarily abnormal because this is also observed in IVF pig embryos. However, the slower rate for CTCF-anchored chromatin loop establishment after ZGA in the uniparental pig embryos compared with IVF pig embryos might be a concern. Further investigation is needed to more fully understand the actual cause of this abnormality.
