# Supplementary material for: The asynchronous establishment of chromatin 3D architecture between in vitro fertilized and uniparental preimplantation pig embryos
Source: Genome Biol. 2020 Aug 10;21:203. doi: 10.1186/s13059-020-02095-z (PMC7418210; doi:10.1186/s13059-020-02095-z)
Supplement: Supplementary file 2 — Additional file 2: Fig. S1. Reproducibility of biological replicates and conservation of chromatin architecture between PEF and MEF. Fig. S2. The prevalence of superdomains in pig preimplantation embryos. Fig. S3. Compartmentalization saddle plots of pig IVF and mouse Hi-C data. Fig. S4. Gradual establishment of TADs during pig IVF embryogenesis. Fig. S5. Loop reprogramming in pig and mouse preimplantation embryos. Fig. S6. Normalized Hi-C contact heatmaps for the same 10 Mb region as Fig. 3a for both replicates. Fig. S7. PC1 values and correlation matrix of chromosome 14 for both replicates and compartmentalization saddle plots of pig PA and AG Hi-C data. Fig. S8. Pearson correlation coefficient of the PC1 using single replicates. Fig. S9. PC1 and correlation matrices of all chromosomes for pig PA and AG embryos at different stages. Fig. S10. TADs are asynchronously established in the two parental alleles. Fig. S11. Embryonic development-related genes enriched in TAD boundaries with different insulation strengths compared uniparental with IVF embryos. [file 13059_2020_2095_MOESM2_ESM.pdf]

# Supplementary Figures

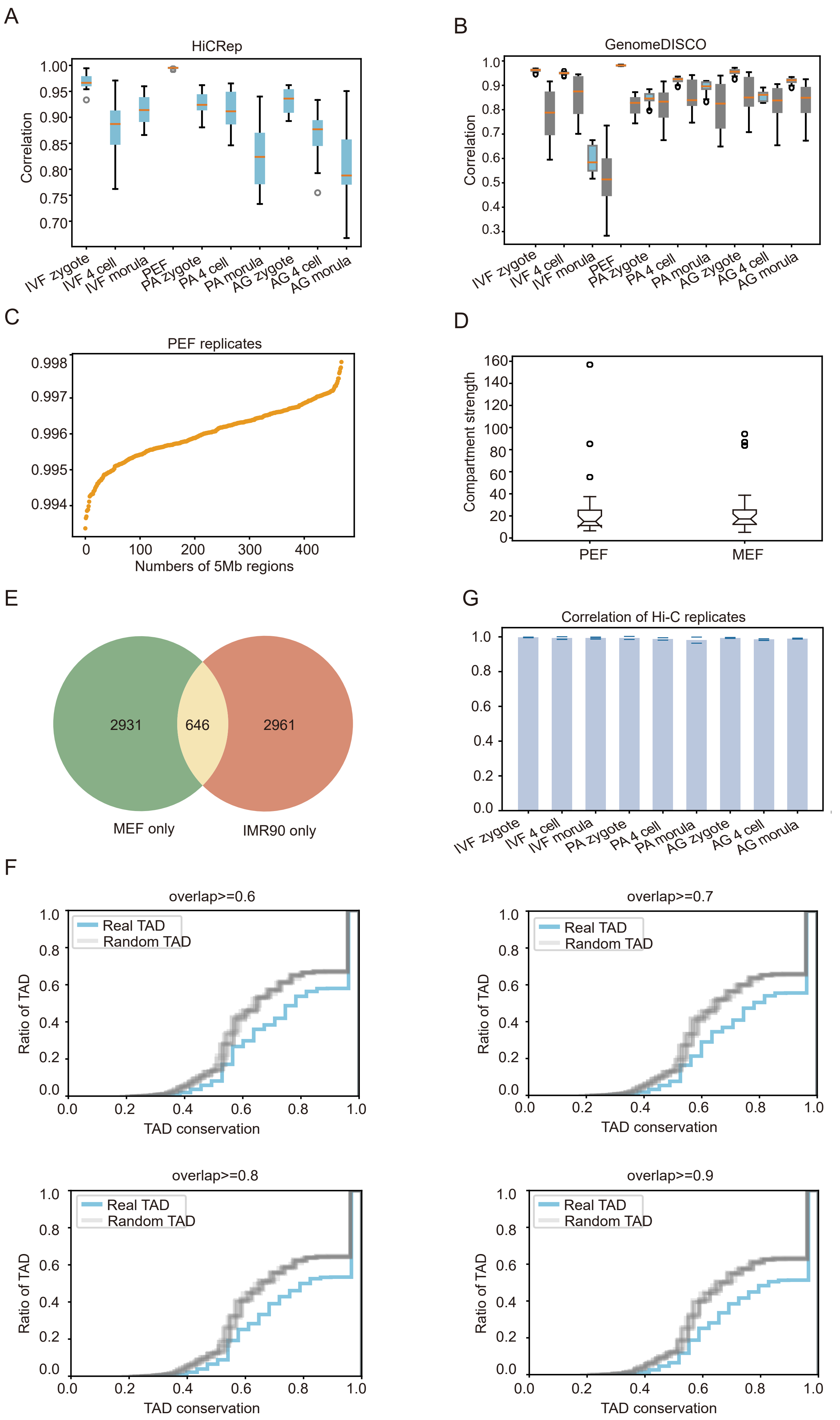

**Fig. S1.** Reproducibility of biological replicates and conservation of chromatin architecture between PEF and MEF. **A**, Reproducibility of biological replicates calculated using HiCRep. **B**, Reproducibility of biological replicates calculated using GenomeDISCO. Correlation between the examined sample and one randomly picked sample was used as control and shown in grey. **C**, Scatter plot showing Pearson's correlation coefficient (PCC) of two PEF Hi-C replicates in every 5Mb sliding windows. **D**, Boxplots showing the compartment strength in PEF and MEF. **E**, Venn graphs showing the overlap of TAD borders between MEF and IMR90 ($P < 4.09E-33$ compared to random, Fisher's exact test). **F**, Cumulative curve of TAD according to TAD conservation. The same ratio under randomly shuffled TADs was shown in gray as control. Four panels were drawn for different overlapping ratio of a bin belonging to one TAD. **G**, Bar charts showing the Pearson's correlation coefficient (PCC) between the two replicates of Hi-C data for early embryos.

A

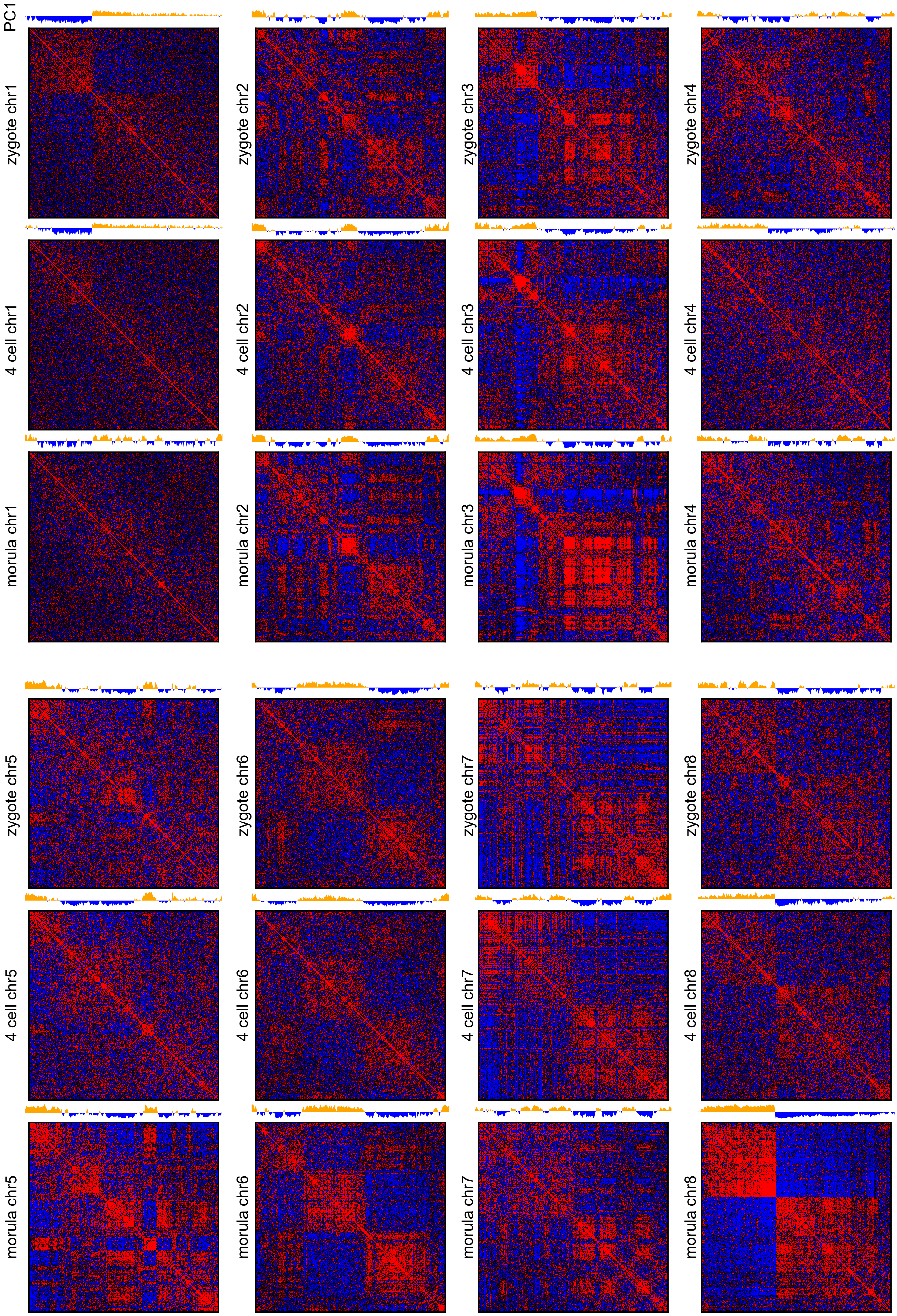

PC1
zygote chr1
zygote chr2
zygote chr3
zygote chr4
4 cell chr1
4 cell chr2
4 cell chr3
4 cell chr4
morula chr1
morula chr2
morula chr3
morula chr4
zygote chr5
zygote chr6
zygote chr7
zygote chr8
4 cell chr5
4 cell chr6
4 cell chr7
4 cell chr8
morula chr5
morula chr6
morula chr7
morula chr8

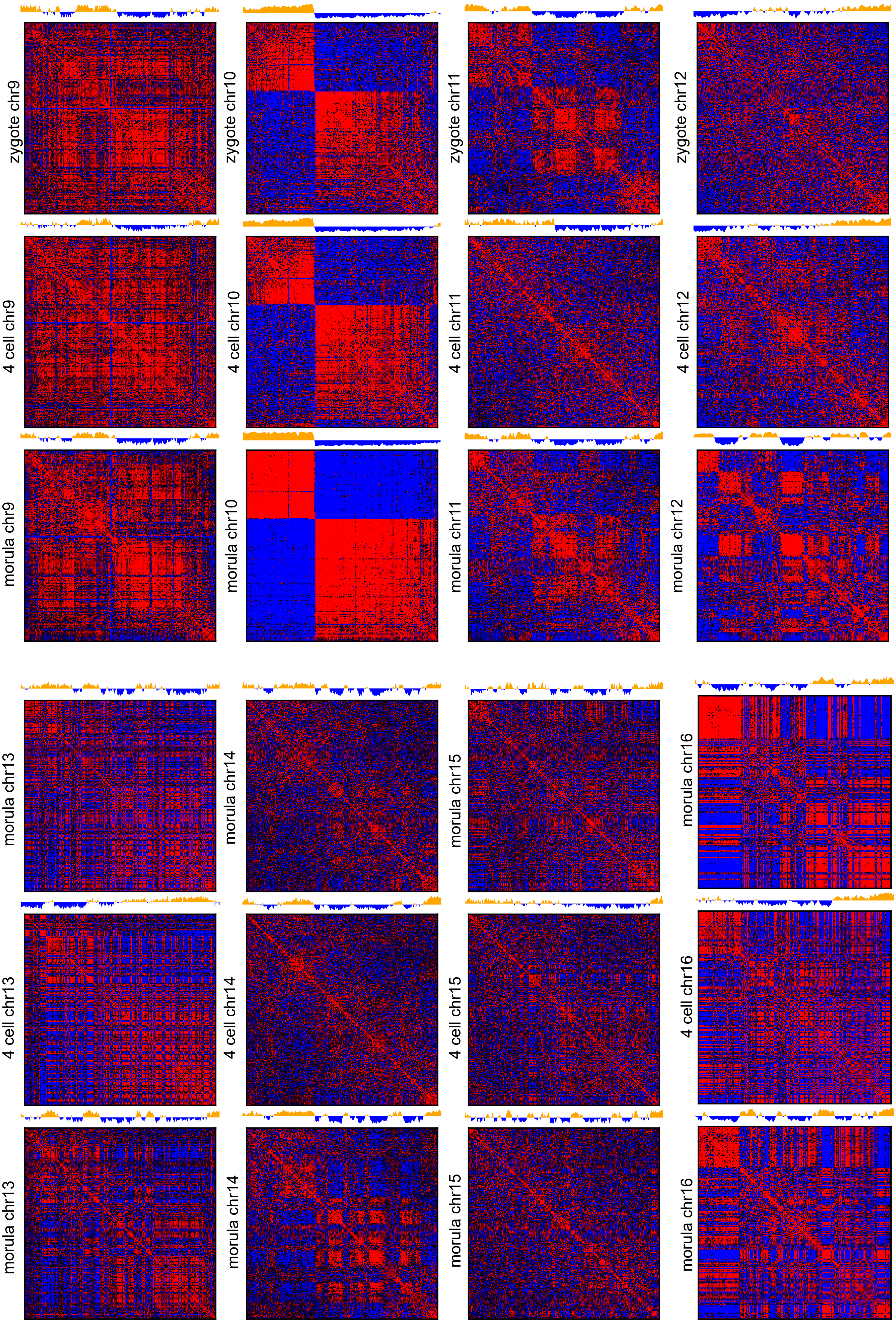
zygote chr9
zygote chr10
zygote chr11
zygote chr12
4 cell chr9
4 cell chr10
4 cell chr11
4 cell chr12
morula chr9
morula chr10
morula chr11
morula chr12
morula chr13
morula chr14
morula chr15
morula chr16
4 cell chr13
4 cell chr14
4 cell chr15
4 cell chr16
morula chr13
morula chr14
morula chr15
morula chr16

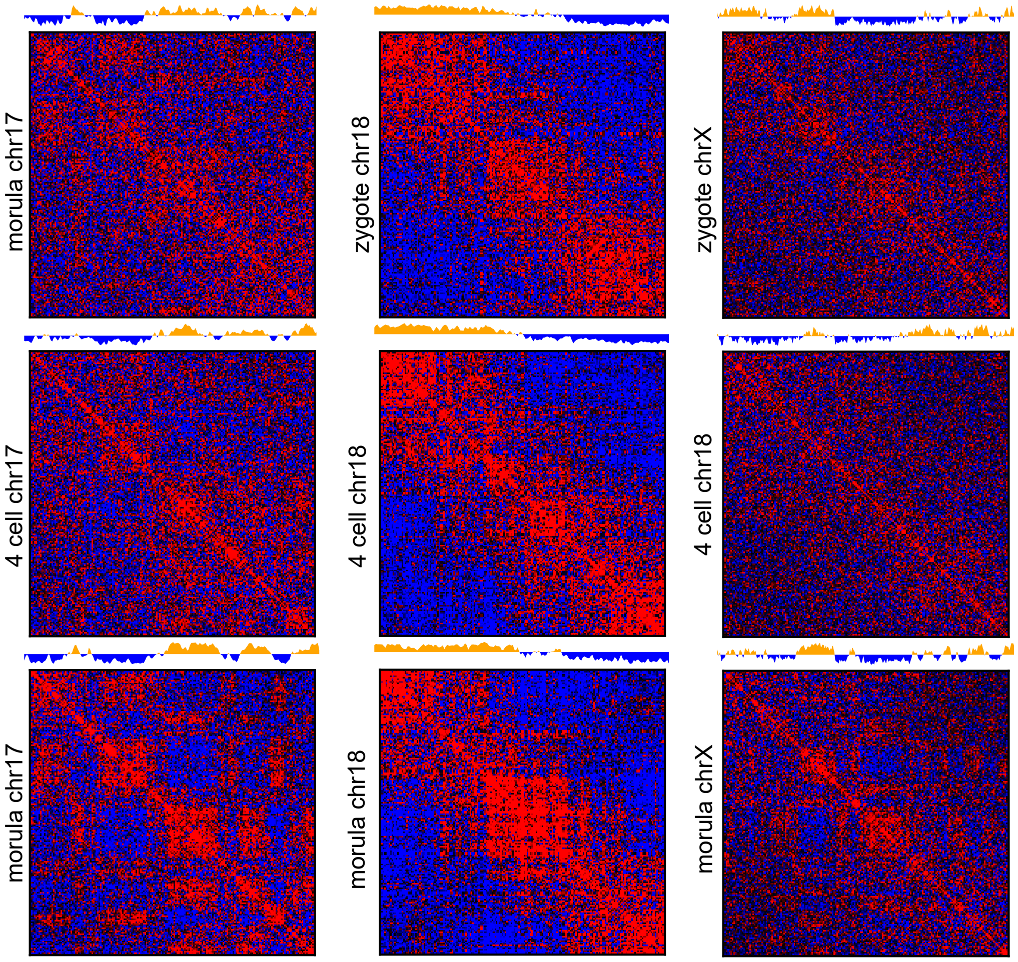


B

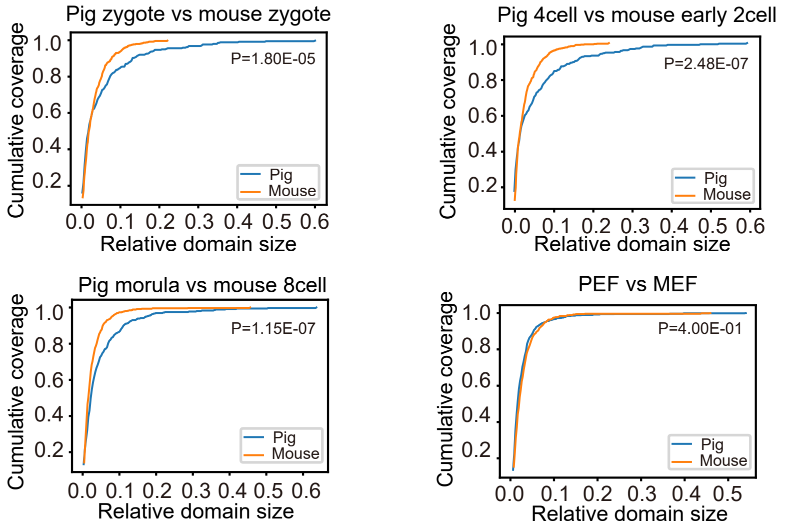


**Fig. S2.** The prevalence of superdomains in pig preimplantation embryos. **A**, PC1 and correlation matrix of all chromosomes for pig IVF embryos. **B**, The accumulative curve for genome coverage by domains size using total raw data. X-axis represents the relative domain size, ie. domain length /chromosome length.

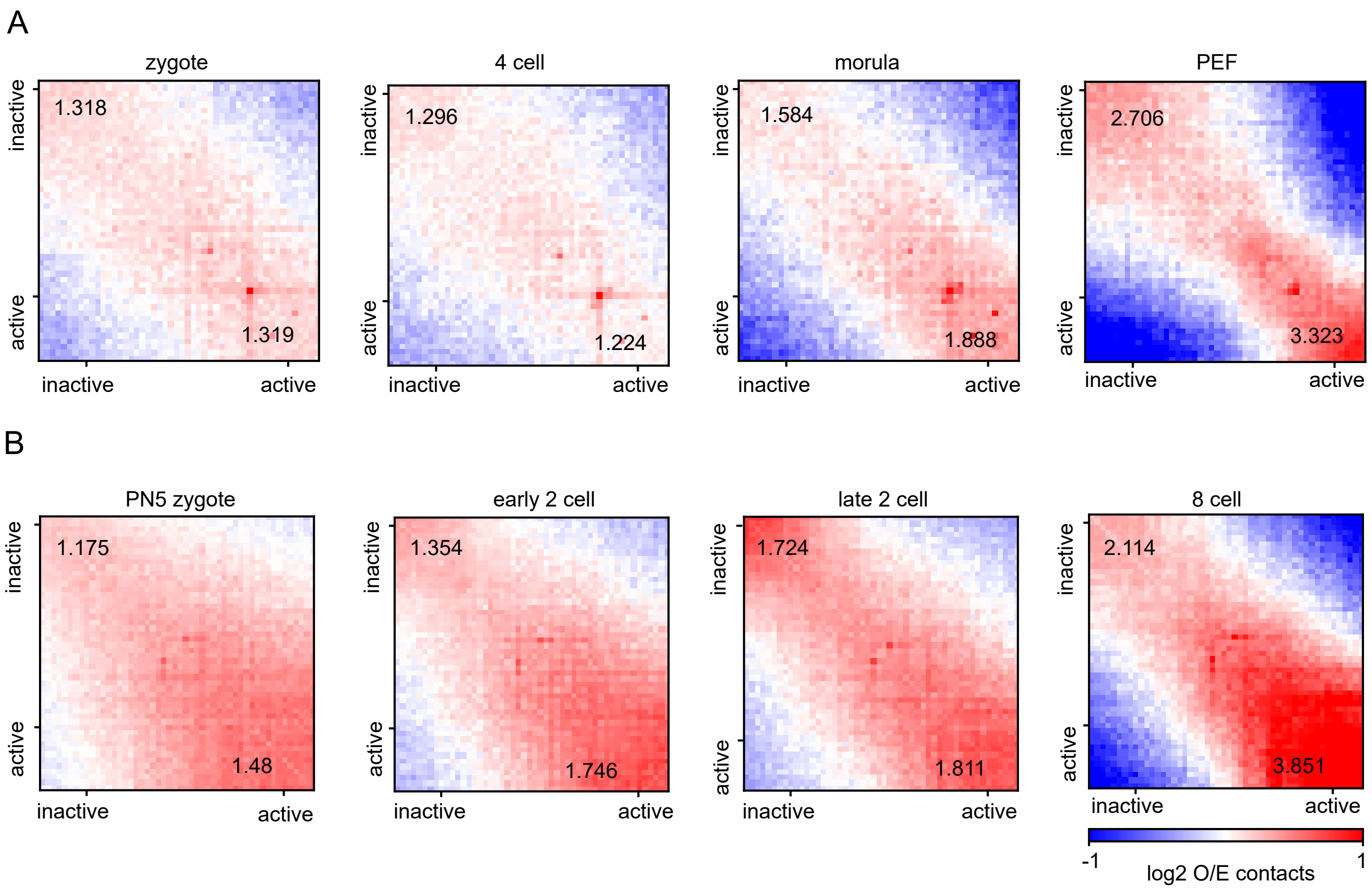


**Fig. S3.** Compartmentalization saddle plots of pig IVF **(A)** and mouse **(B)** Hi-C data binned at 300kb resolution at different developmental stages. For embryos, bins are sorted by PC1 value derived from Hi-C data obtained with morula and 8 cell for pig and mouse, respectively. For PEF, the PC1 value of itself was used to sorted bins. In these plots preferential B-B interactions are in the upper left corner, and preferential A-A interactions are in the lower right corner. Numbers in corners represent the strength of AA interactions as compared to AB interaction and BB interactions over BA interactions.

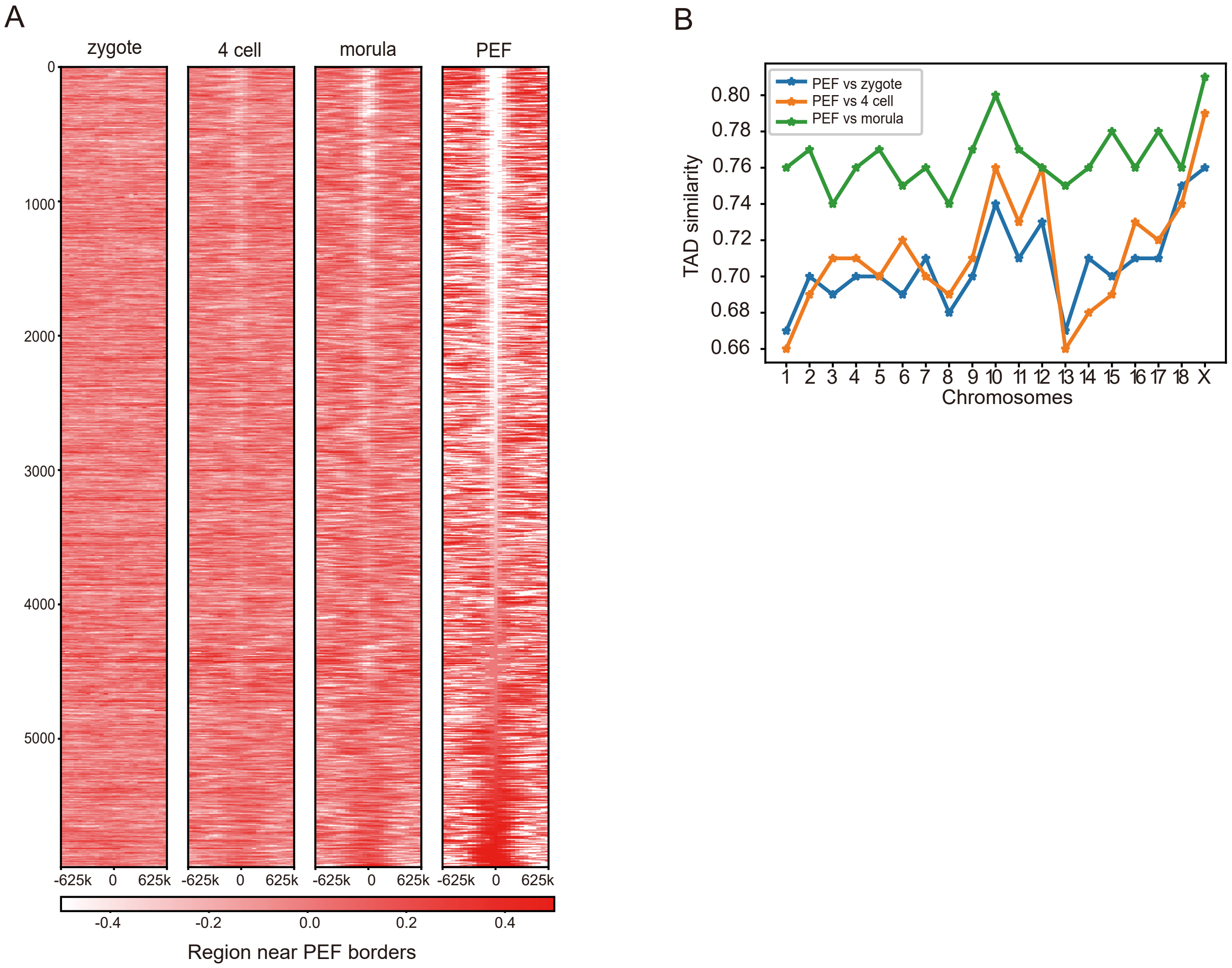


**Fig. S4.** Gradual establishment of TADs during pig IVF embryogenesis. **A,** Heatmaps showing the strength of all TAD boundaries at different developmental stages of pig IVF embryos. **B,** TAD similarity between PEF and developmental embryos of IVF for each chromosome.

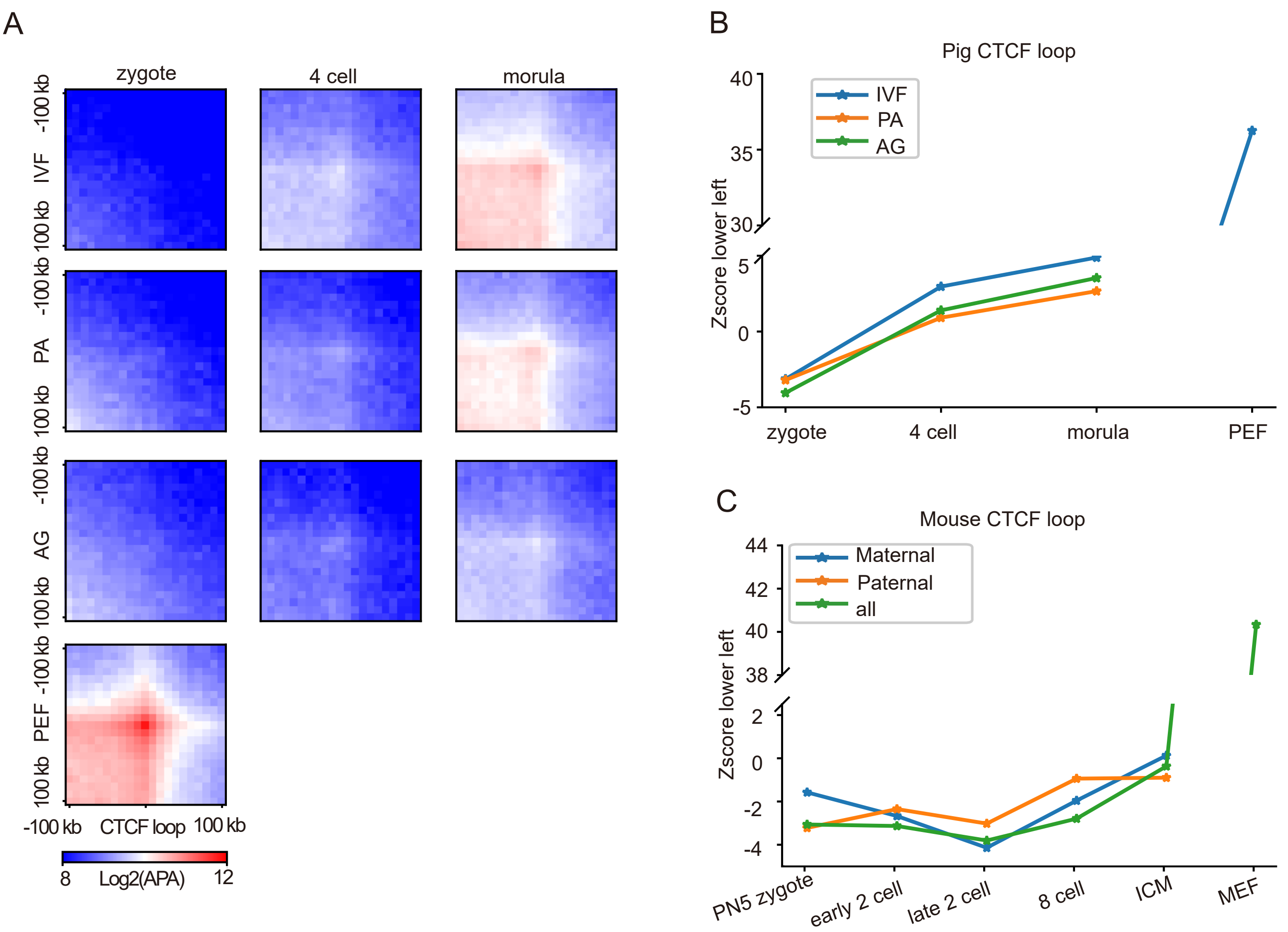


**Fig. S5.** Loop reprogramming in pig and mouse preimplantation embryos. **A,** Aggregate loop plots showing the strength of PEF CTCF loops in IVF, PA and AG preimplantation embryos and in PEF. **B,** Z-score of the central pixel relative to the all of the pixels in the lower left corner for pig embryonic samples. **C,** Z-score of MEF CTCF loops in each stage of mouse embryogenesis for maternal and paternal alleles. The score was also shown for all reads.

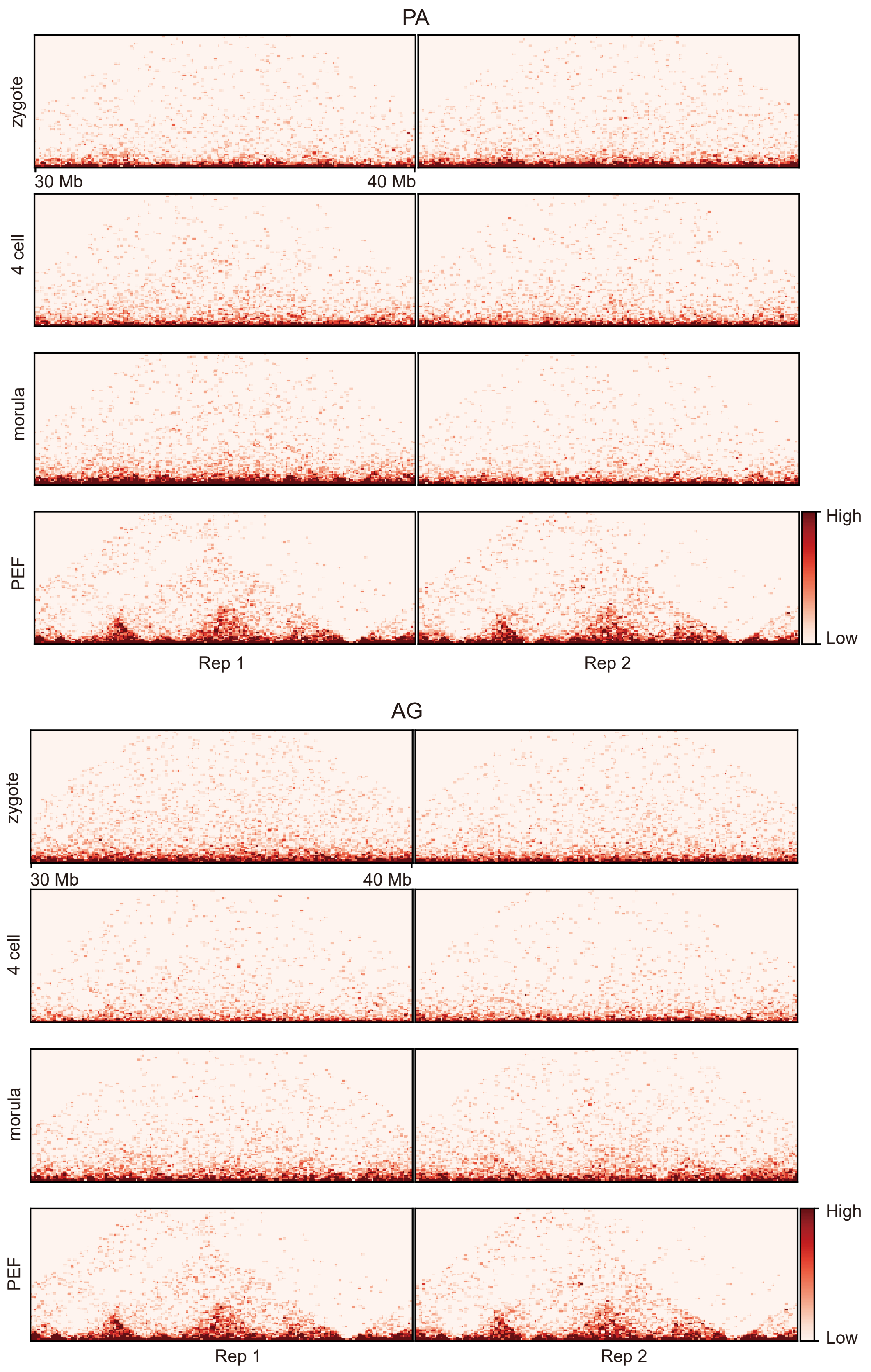


**Fig. S6.** Normalized Hi-C contact heatmaps for the same 10 Mb region as figure3A for both replicates.

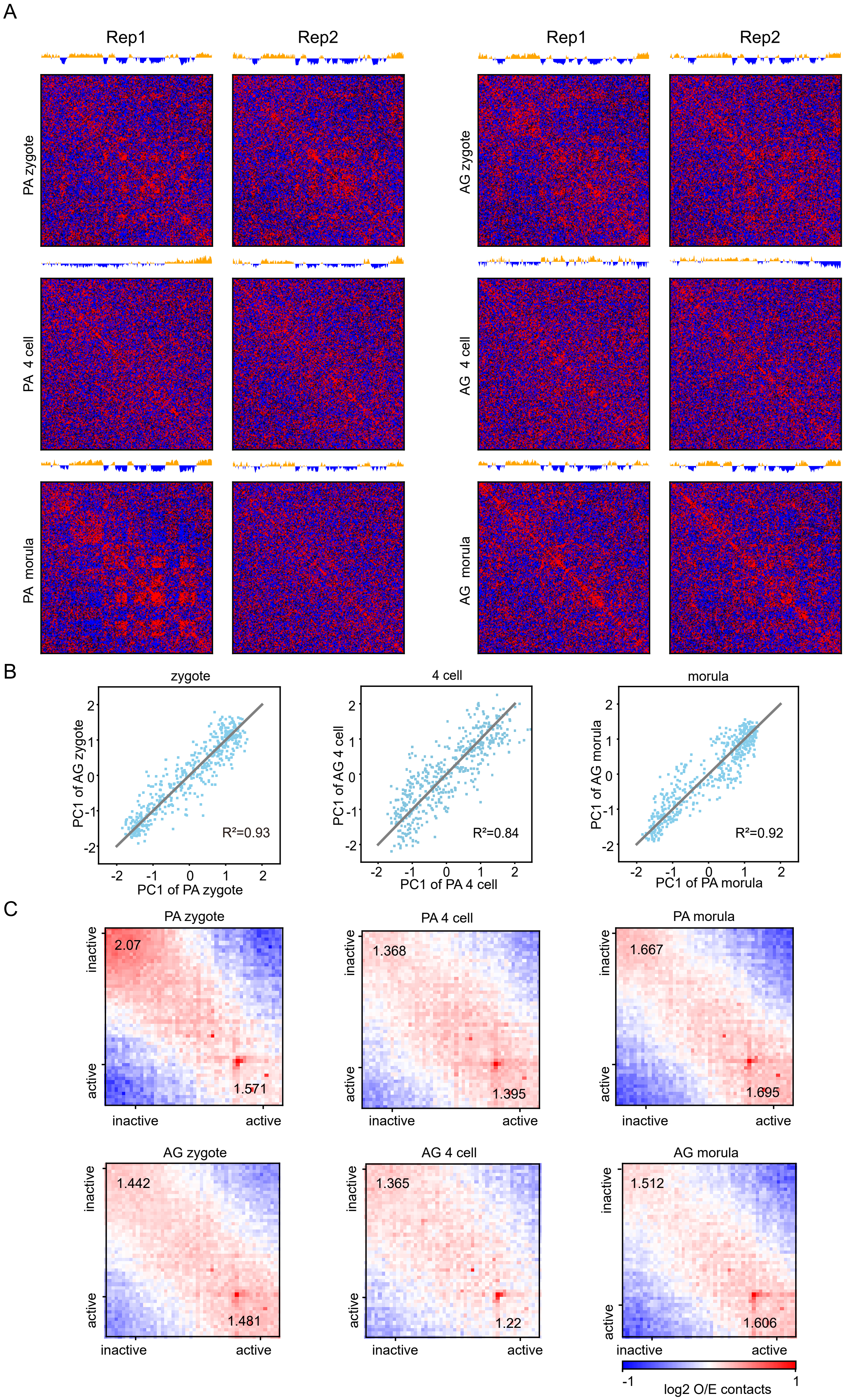

A
Rep1
Rep2
Rep1
Rep2
PA zygote
AG zygote
PA 4 cell
AG 4 cell
PA morula
AG morula
B
zygote
PC1 of AG zygote
PC1 of PA zygote
R²=0.93
4 cell
PC1 of AG 4 cell
PC1 of PA 4 cell
R²=0.84
morula
PC1 of AG morula
PC1 of PA morula
R²=0.92
C
PA zygote
2.07
1.571
PA 4 cell
1.368
1.395
PA morula
1.667
1.695
AG zygote
1.442
1.481
AG 4 cell
1.365
1.22
AG morula
1.512
1.606
inactive
active
log2 O/E contacts
-1
1

D

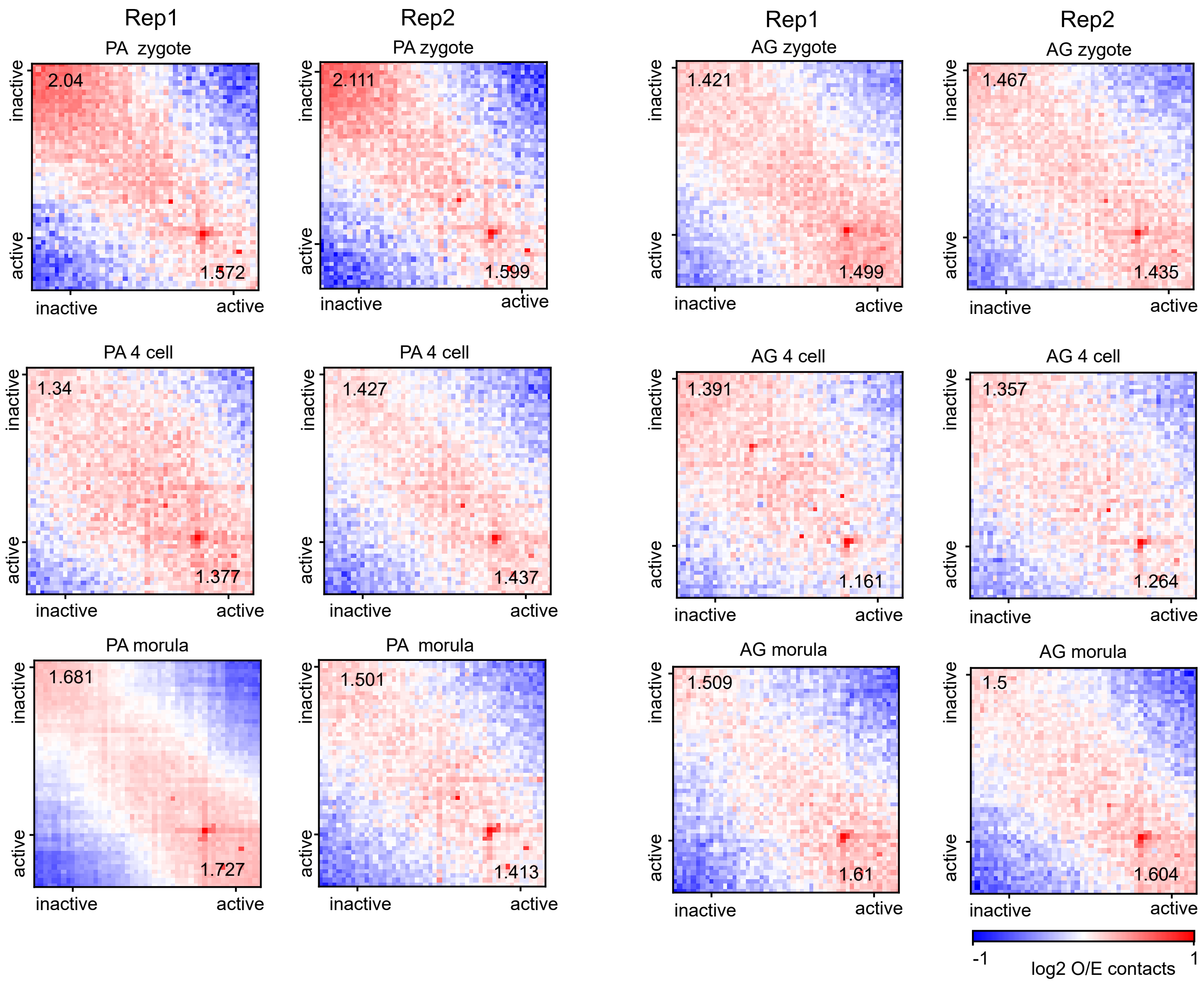


**Fig. S7.** PC1 values and correlation matrix of chromosome 14 for both replicates and compartmentalization saddle plots of pig PA and AG Hi-C data. **A**, PC1 values and correlation matrix of chromosome 14 for both replicates. **B**, Scatter plots of the compartments scores in chromosome 14. Each dot represents the PC1 value of a bin (binsize=300kb). **C**, Compartmentalization saddle plots of pig PA and AG Hi-C data binned at 300kb resolution at different developmental stages. Bins are sorted by PC1 value derived from Hi-C data obtained with IVF morula. **D**, Compartmentalization saddle plots for both replicates.

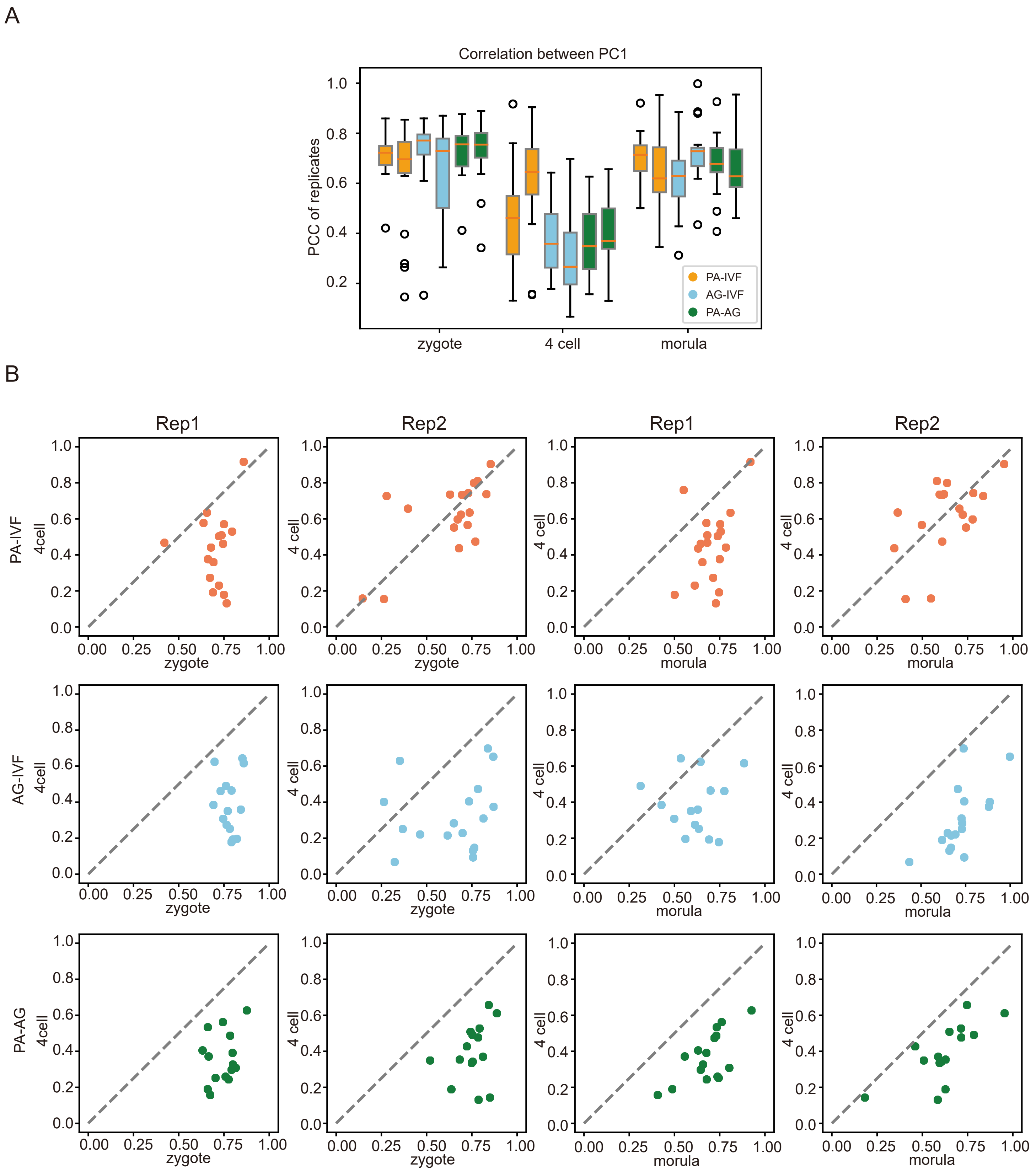


**Fig. S8.** Boxplots and scatter plots showed Pearson correlation coefficient of the PC1 using single replicates.

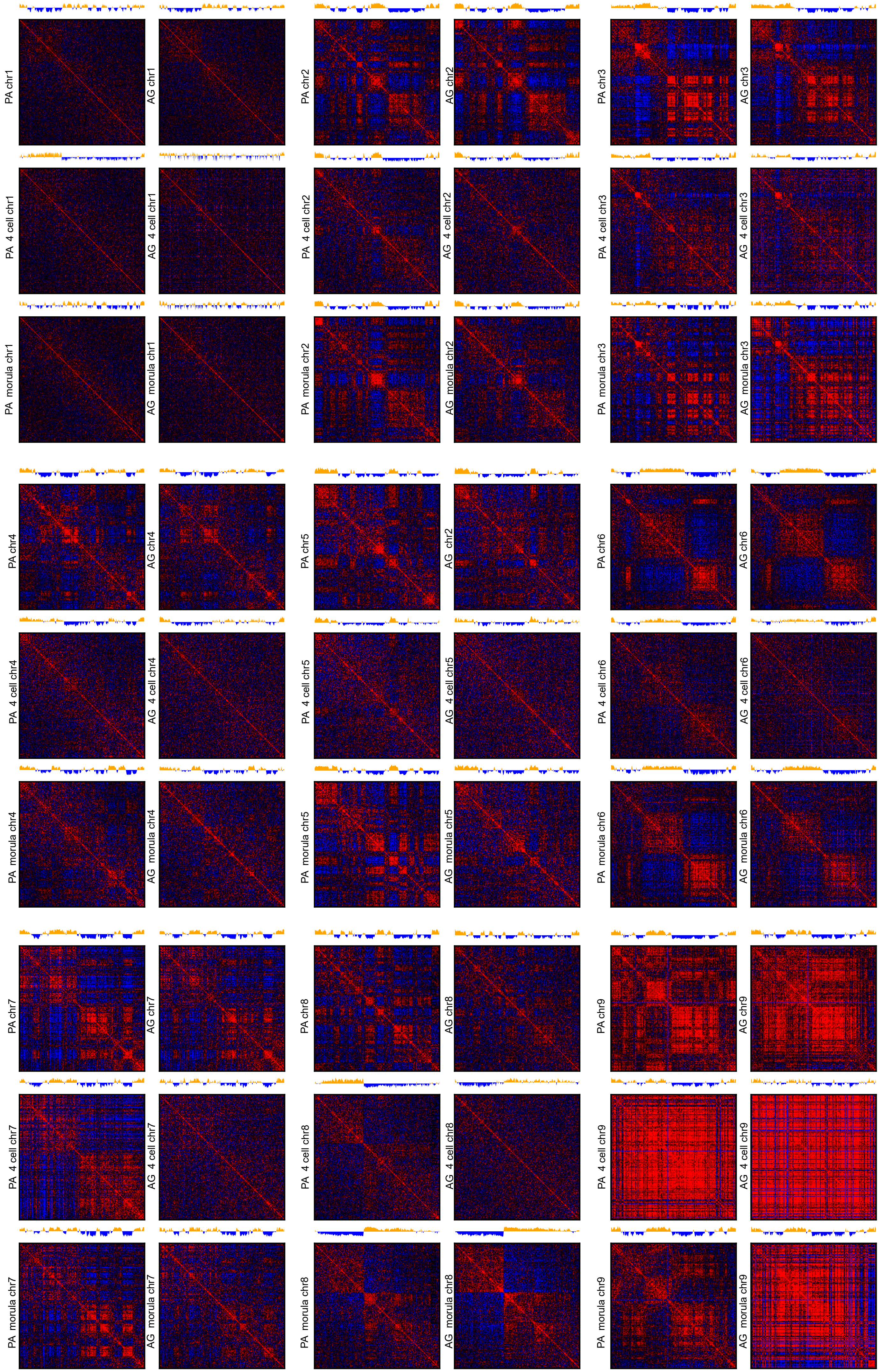
PA chr1
AG chr1
PA chr2
AG chr2
PA chr3
AG chr3
PA 4 cell chr1
AG 4 cell chr1
PA 4 cell chr2
AG 4 cell chr2
PA 4 cell chr3
AG 4 cell chr3
PA morula chr1
AG morula chr1
PA morula chr2
AG morula chr2
PA morula chr3
AG morula chr3
PA chr4
AG chr4
PA chr5
AG chr2
PA chr6
AG chr6
PA 4 cell chr4
AG 4 cell chr4
PA 4 cell chr5
AG 4 cell chr5
PA 4 cell chr6
AG 4 cell chr6
PA morula chr4
AG morula chr4
PA morula chr5
AG morula chr5
PA morula chr6
AG morula chr6
PA chr7
AG chr7
PA chr8
AG chr8
PA chr9
AG chr9
PA 4 cell chr7
AG 4 cell chr7
PA 4 cell chr8
AG 4 cell chr8
PA 4 cell chr9
AG 4 cell chr9
PA morula chr7
AG morula chr7
PA morula chr8
AG morula chr8
PA morula chr9
AG morula chr9

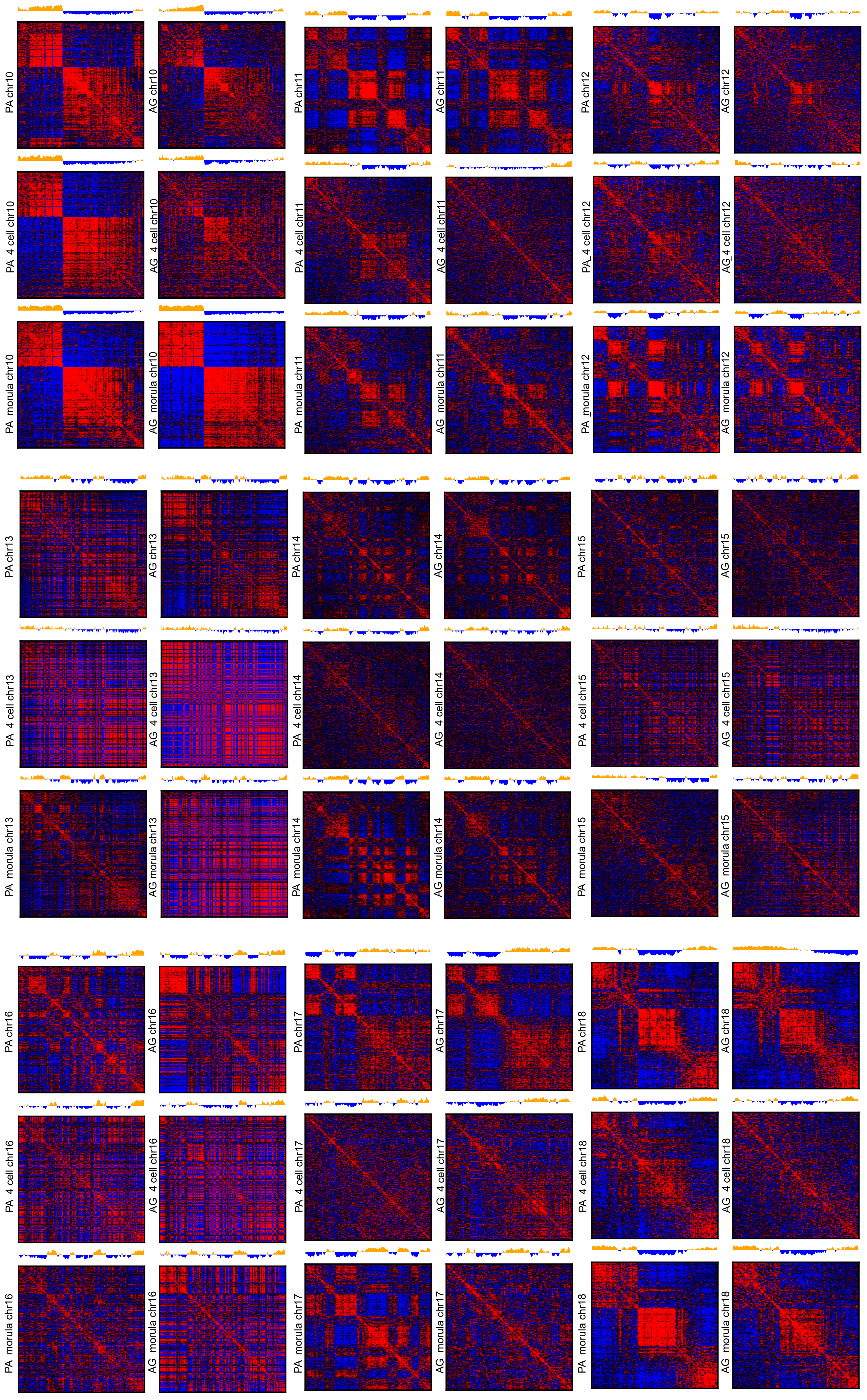
PA chr10
AG chr10
PA chr11
AG chr11
PA chr12
AG chr12
PA 4 cell chr10
AG 4 cell chr10
PA 4 cell chr11
AG 4 cell chr11
PA_4 cell chr12
AG_4 cell chr12
PA morula chr10
AG morula chr10
PA morula chr11
AG morula chr11
PA_morula chr12
AG morula chr12
PA chr13
AG chr13
PA chr14
AG chr14
PA chr15
AG chr15
PA 4 cell chr13
AG 4 cell chr13
PA 4 cell chr14
AG 4 cell chr14
PA 4 cell chr15
AG 4 cell chr15
PA morula chr13
AG morula chr13
PA morula chr14
AG morula chr14
PA morula chr15
AG morula chr15
PA chr16
AG chr16
PA chr17
AG chr17
PA chr18
AG chr18
PA 4 cell chr16
AG 4 cell chr16
PA 4 cell chr17
AG 4 cell chr17
PA 4 cell chr18
AG 4 cell chr18
PA morula chr16
AG morula chr16
PA morula chr17
AG morula chr17
PA morula chr18
AG morula chr18

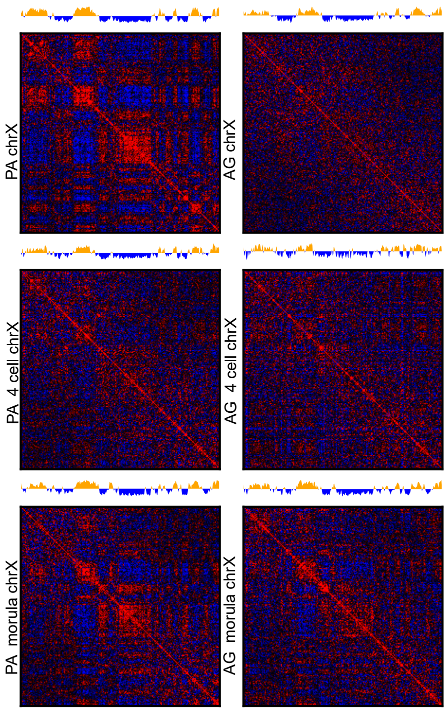


**Fig. S9.** PC1 and correlation matrices of all chromosomes for pig PA and AG embryos at different stages.

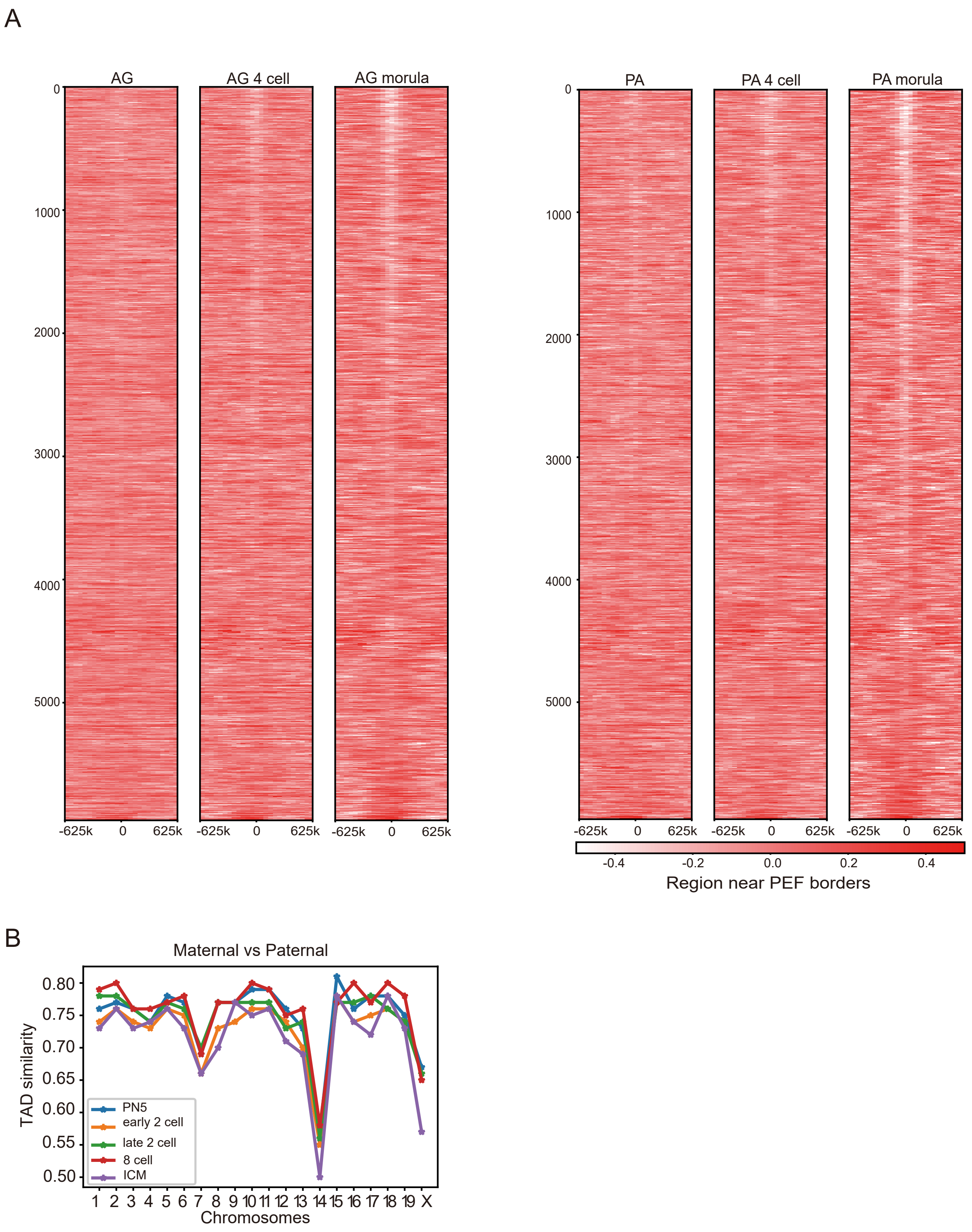


**Fig. S10.** TADs are asynchronously established in the two parental alleles. **A**, Heatmaps showing the strength of all TAD boundaries at different developmental stages of pig PA and AG embryos. **B**, TAD similarity between maternal and paternal alleles in different developmental stages of mouse embryogenesis.

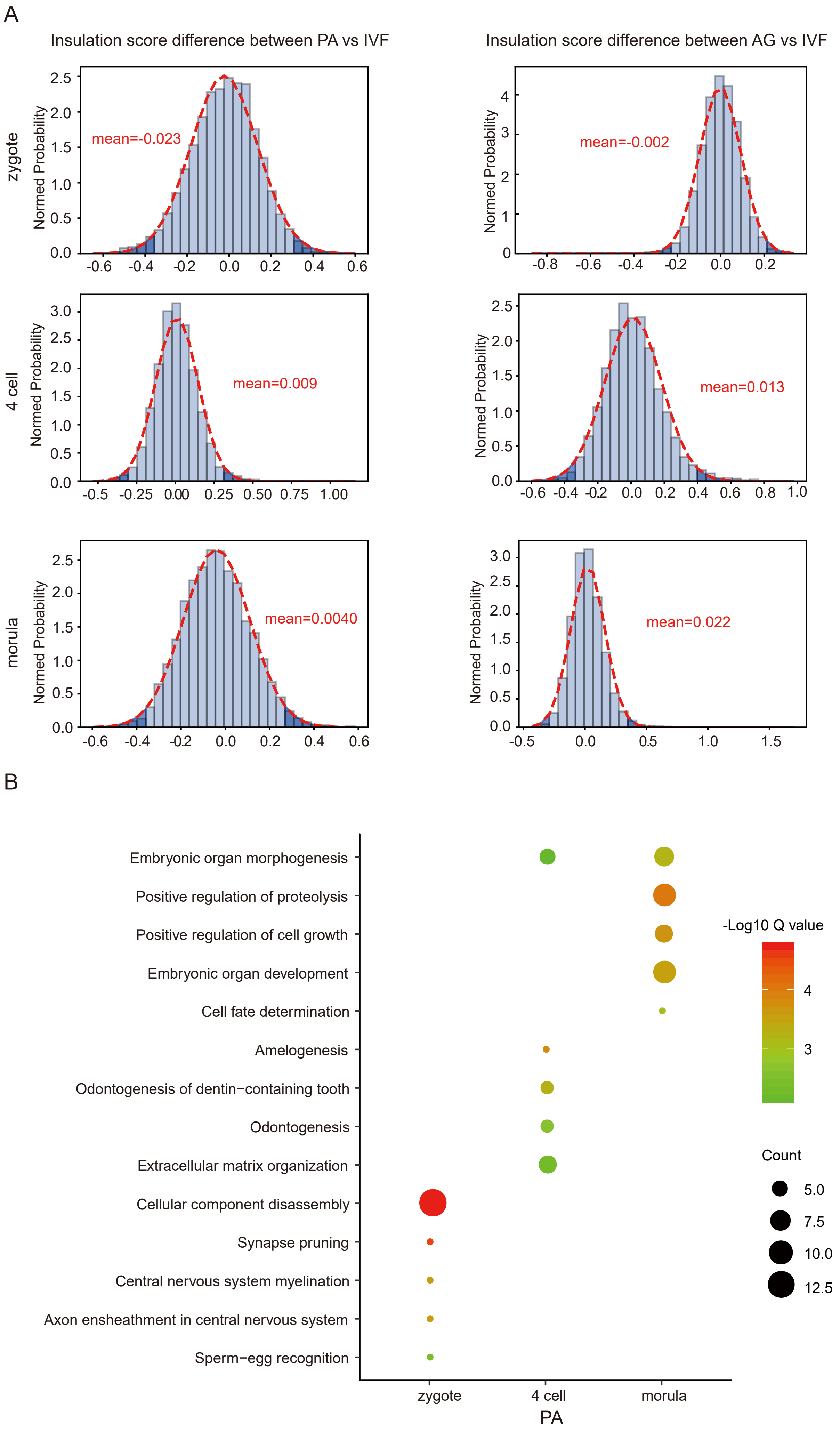
A
Insulation score difference between PA vs IVF
Insulation score difference between AG vs IVF
zygote
4 cell
morula
Normed Probability
mean=-0.023
mean=-0.002
mean=0.009
mean=0.013
mean=0.0040
mean=0.022
B
Embryonic organ morphogenesis
Positive regulation of proteolysis
Positive regulation of cell growth
Embryonic organ development
Cell fate determination
Amelogenesis
Odontogenesis of dentin−containing tooth
Odontogenesis
Extracellular matrix organization
Cellular component disassembly
Synapse pruning
Central nervous system myelination
Axon ensheathment in central nervous system
Sperm−egg recognition
zygote
4 cell
morula
PA
-Log10 Q value
4
3
Count
5.0
7.5
10.0
12.5

C

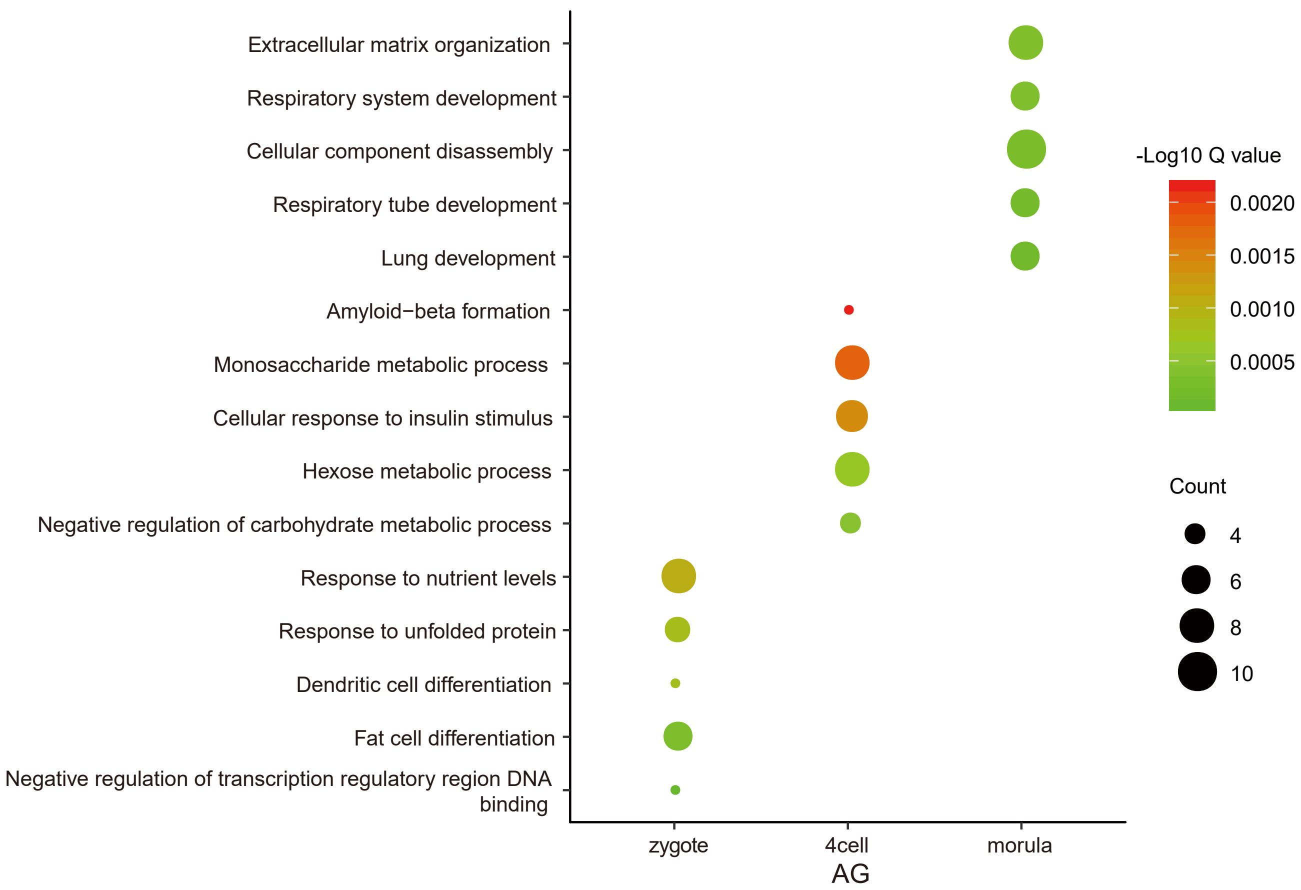


**Fig. S11.** Embryonic development-related genes enriched in TAD boundaries with different insulation strengths compared uniparental with IVF embryos. **A**, Left: Histograms showing the IS difference of PA borders and IVF borders in 1cell, 4cell and morula stage. 5% confidence intervals are colored in dark blue. Right: IS difference of AG borders and IVF borders. **B**, **C**, The enriched GO terms for genes that are located within TAD borders having significantly different insulation strength between PA and IVF embryos (B) as well as between AG and IVF embryos (C).
