# Supplementary material for: The asynchronous establishment of chromatin 3D architecture between in vitro fertilized and uniparental preimplantation pig embryos
Source: Genome Biol. 2020 Aug 10;21:203. doi: 10.1186/s13059-020-02095-z (PMC7418210; doi:10.1186/s13059-020-02095-z)
Supplement: Supplementary file 4 — Additional file 4: Table S2. Comparison of the p(s) curve between uniparental embryos and the IVF embryos. [file 13059_2020_2095_MOESM4_ESM.docx]

Table S2. Comparison of the p(s) curve between uniparental embryos and the IVF embryos

| Large than 10 MB | | | | |
| --- | --- | --- | --- | --- |
| samples | JSD of Uniparental and IVF | Mean JSD of replicates | P value | P value <0.05 |
| PA_zyg | 0.0029 | 0.0005 | 1.5e-06 | √ |
| PA_4cell | 0.0003 | 0.0005 | 0.377 |  |
| PA_morula | 0.0004 | 0.0005 | 0.700 |  |
| AG_zyg | 0.0008 | 0.0005 | 0.155 |  |
| AG_4cell | 0.0010 | 0.0005 | 0.030 | √ |
| AG_morula | 0.0013 | 0.0005 | 0.004 | √ |

| 50KB ~ 1 MB | | | | |
| --- | --- | --- | --- | --- |
| samples | JSD of Uniparental and IVF | Mean JSD of replicates | P value | P value <0.05 |
| PA_zyg | 0.0056 | 0.0006 | 0.0 | √ |
| PA_4cell | 0.0024 | 0.0006 | 2.94e-05 | √ |
| PA_morula | 0.0023 | 0.0006 | 6.84e-05 | √ |
| AG_zyg | 0.0005 | 0.0006 | 0.491 |  |
| AG_4cell | 0.0018 | 0.0006 | 0.0008 | √ |
| AG_morula | 0.0001 | 0.0006 | 0.074 |  |
